# Comparative efficacy and safety of acupuncture for adolescent depression: protocol for a systematic review and Bayesian network meta-analysis

**DOI:** 10.3389/fpsyt.2025.1624825

**Published:** 2025-07-23

**Authors:** Jiayu Zhang, Jiafeng Wang, Xingyu Xiang, Na Zhang, Kaiying Zhang, Pengcheng Zhang, Hao Wen

**Affiliations:** ^1^ Guangzhou Health Science College, Guangzhou, China; ^2^ Guangzhou Huangpu District Hospital of Traditional Chinese Medicine, Guangzhou, China; ^3^ The Affiliated Traditional Chinese Medicine Hospital of Guangzhou Medical University, Guangzhou, China; ^4^ Guangzhou University of Chinese Medicine, Guangzhou, China

**Keywords:** adolescent depression, acupuncture, network meta-analysis, protocol, systematic review

## Abstract

**Background:**

Adolescents are in a critical stage of development and represent a high-risk population for depression. Various studies have demonstrated the efficacy of acupuncture therapies in the treatment of adolescent depression. However, trials directly comparing the efficacy and safety of different acupuncture therapies for adolescent depression are still lacking. To identify the optimal acupuncture therapy for treating adolescent depression, we will conduct a systematic review and network meta-analysis of various acupuncture therapies for adolescent depression.

**Methods:**

Randomized controlled trials (RCTs) investigating the efficacy and safety of acupuncture in adolescent depression will be systematically searched across the Cochrane Central Register of Controlled Trials (CENTRAL), PubMed, Embase, Web of Science, and the China National Knowledge Infrastructure (CNKI) databases from inception to December 2025. Study quality will be assessed using the Cochrane Risk of Bias Tool (RoB 2.0), while the Confidence in Network Meta-Analysis (CINeMA) framework will evaluate evidence certainty. The primary outcomes will be included at least one validated depression rating scale for adolescent. Secondary endpoints include safety outcomes, measured by the incidence of adverse events during the study period. Bayesian network meta-analysis will be performed using the gemtc package (v0.8-7) in R statistical software (version 4.0.5).

**Discussion:**

This study will clarify the comparative efficacy and safety of acupuncture for adolescent depression, guiding clinical decision-making.

**Prospero Registration Number:**

identifier (CRD 42024581768).

## 1 Introduction

Depression is a complex mental health disorder characterized by a persistent lack of interest in daily activities, accompanied by a range of emotional, cognitive, physical, and behavioral symptoms (1). It is recognized as one of the leading causes of disability worldwide (2). Depression significantly impairs the quality of life for patients and their families while imposing a substantial economic burden on society (3). Adolescents are in a critical stage of development and represent a high-risk population for depression (4). Compared to late-onset depression, early-onset depression in this group often leads to more severe consequences, including substance abuse, physical illnesses, social functional impairment, and even self-harm or suicidal behaviors (5, 6). Additionally, young individuals with depression are more likely to experience a higher tendency of recurrence (5).

Currently, pharmacotherapy and psychotherapy are the dominant treatments for depression. However, long-term antidepressant use is associated with significant side effects (7). Despite advancements in modern medicine and the emergence of novel antidepressants that have improved treatment efficacy, these medications predominantly target adult populations. In contrast, the development of specific pharmacological interventions for adolescent depression remains an area of active exploration (8, 9). Prior studies have shown that certain antidepressants may elevate suicide risk in adolescents, highlighting the need for stringent safety assessments when selecting antidepressant therapies for this vulnerable population. It is crucial to balance symptom alleviation with the avoidance of adverse effects that could worsen the condition or cause additional harm (10). Therefore, there is an urgent need to identify safe and side-effect-free therapeutic strategies for adolescent depression.

Acupuncture, a widely accepted complementary and alternative medical practice, has gained global popularity (11). Electroacupuncture represents a contemporary evolution of traditional acupuncture, distinguished by the application of controlled electrical currents (typically 2-100 Hz) to inserted needles. A clinical trial involving 60 depressed patients demonstrated that electroacupuncture improved overall clinical impression, anxiety/somatization, and feelings of hopelessness more effectively than selective serotonin reuptake inhibitors (SSRIs) (12). Animal experiments conducted by some researchers revealed that acupuncture significantly reversed depressive-like behaviors in model rats during open-field tests and reduced abnormally elevated glutamate (Glu) levels in the hippocampus (13). Furthermore, several RCTs have shown that acupuncture may serve as a safe adjunctive therapy to antidepressant medications while exhibiting greater efficacy in alleviating depressive symptoms (14–16). Despite the increasing number of studies, the efficacy of acupuncture in treating adolescent depression remains controversial due to the heterogeneity in study designs, inconsistencies in quality control, and methodological limitations across existing research. Network meta-analysis (NMA) is a method that allows for the simultaneous comparison of multiple treatments within a single meta-analysis, thus identifying the potentially optimal treatment choice (17). Therefore, we have developed a protocol for an NMA to evaluate the efficacy and safety of various acupuncture therapies for adolescent depression. This protocol outlines systematic methods for assessing clinical trial data to identify the most effective acupuncture techniques.

## 2 Methods

### 2.1 Study registration

This systematic review and network meta-analysis is conducted rigorously following the PRISMA 2020, which has been registered in the International Prospective Register of Systematic Reviews (PROSPERO; registration number: CRD42024581768). The study protocol followed the guidelines of the Preferred Reporting Items for Systematic Reviews and Meta-Analyses Protocols statement (PRISMA-P) (18), which provided in (**Supplementary Materials S1**). Any protocol amendments occurring during the review process will be transparently documented in the final manuscript.

### 2.2 Eligibility criteria

#### 2.2.1 Types of studies

Only RCTs reported in English or Chinese will be included, without geographical limitations. Non-randomized trials, observational studies, reviews, animal experimental studies, case studies, expert opinions, and studies with unavailable full-text will be excluded.

#### 2.2.2 Types of participants

We will include adolescents (19) aged 10–19 years who were diagnosed with Diagnostic and Statistical Manual of Mental Disorders (DSM-5), Chinese Classification of Mental Disorders (CCMD-3) or equivalent diagnostic criteria (e.g., Diagnostic and Statistical Manual of Mental Disorders, Fourth Edition (DSM-4), International Classification of Diseases, Tenth/Eleventh Revision (ICD10/11)). Participants will be included regardless of gender or disease duration.

#### 2.2.3 Types of interventions

Acupuncture is defined as any acupoint-based therapy, regardless of the needling or stimulation techniques. Eligible interventions include single or combined antidepressants, as well as acupuncture combined with other therapies. Non-acupoint-based interventions will be excluded, as acupuncture operates by stimulating specific points on the body (‘acupoints’) that have been proven to influence brain networks involved in mood regulation. Studies employing random needle placement (e.g., inserting needles at non-acupoint locations) or sham devices (e.g., placebo needles that do not penetrate the skin) are unable to determine whether true acupuncture provides benefits for depression.

#### 2.2.4 Types of comparisons

Guideline-recommended therapies for depression (e.g., Western medication, lifestyle interventions, supplements), combined therapies, different acupuncture methods, and inactive interventions like sham acupuncture, waiting lists, and placebos.

#### 2.2.5 Types of outcome measures

The primary outcomes will be included at least one validated depression rating scale (e.g., the Reynolds Adolescent Depression Scale (RADS-2), the Children’s Depression Inventory (CDI), or Children’s Depression Rating Scale-Revised (CDRS-R)). The secondary endpoint will be safety, quantified by the incidence of adverse reactions during the study period.

### 2.3 Search strategy

We will perform a comprehensive search of the following databases from inception to December 2025: the Cochrane Central Register of Controlled Trials (CENTRAL), PubMed, Embase, Web of Science, and the China National Knowledge Infrastructure (CNKI) database. The search strategies will be exemplified using PubMed, as detailed in **Table 1**. All details of the search strategies will be developed by two independent investigators (see **Supplementary Material S2**). Manual searches will also be conducted on reference lists of retrieved articles and relevant systematic reviews, including but not limited to the following methods: a. Checking reference lists: Reviewing the reference lists of included studies as well as relevant systematic reviews and literature reviews to identify additional potentially relevant research; b. Citation tracking: Using citation tracking tools (e.g., Google Scholar or Web of Science) to locate subsequent citations of known relevant articles, thereby expanding the scope of literature sources.

**Table 1:** Search strategy for PubMed database.

NO.	Search terms
#1	Search (“depression” [MeSH Terms])
#2	Search (“depression” [Title/Abstract] OR “depressive disorder” [Title/Abstract] OR “depressive”[Title/Abstract]
#3	**#1 or #2**
#4	Search (“acupuncture”[MeSH Terms])
#5	Search (“acupuncture”[Title/Abstract] OR “electro-acupuncture” [Title/Abstract] OR “warming needling” [Title/Abstract] OR “fire needling”[Title/Abstract] OR “bloodletting”[Title/Abstract] OR “auriculo-acupuncture”[Title/Abstract] OR “auricular” [Title/Abstract] OR “moxibustion” [Title/Abstract] OR, “cupping” [Title/Abstract]OR “acupoint*” [Title/Abstract])
#6	**#4 or #5**
#7	Search (“adolescent”[MeSH Terms]
#8	“adolescent” [Title/Abstract] OR “teenager” [Title/Abstract] OR “juvenile” [Title/Abstract] OR “young” [Title/Abstract] OR “youngsters”[Title/Abstract] OR “student”[Title/Abstract]
#9	**#7 or #8**
#10	Search (“randomized controlled trial” [Title/Abstract] OR “controlled clinical “[Title/Abstract] OR “trial “[Title/Abstract] OR “group”[Title/Abstract] OR “placebo” [Title/Abstract] OR “randomly” [Title/Abstract])
#11	**#3 and #6 and #9 and #10**

### 2.4 Study selection and data extraction

The literature management process will be facilitated through the use of EndNote X9 and Microsoft Excel. Two independent investigators (XYX and NZ) will conduct the initial literature search. Subsequently, three trained reviewers (JFW, KYZ, and YQS) will perform preliminary screening based on titles and abstracts. Full-text articles meeting the pre-defined eligibility criteria will be retrieved for comprehensive evaluation.

**Figure 1:**
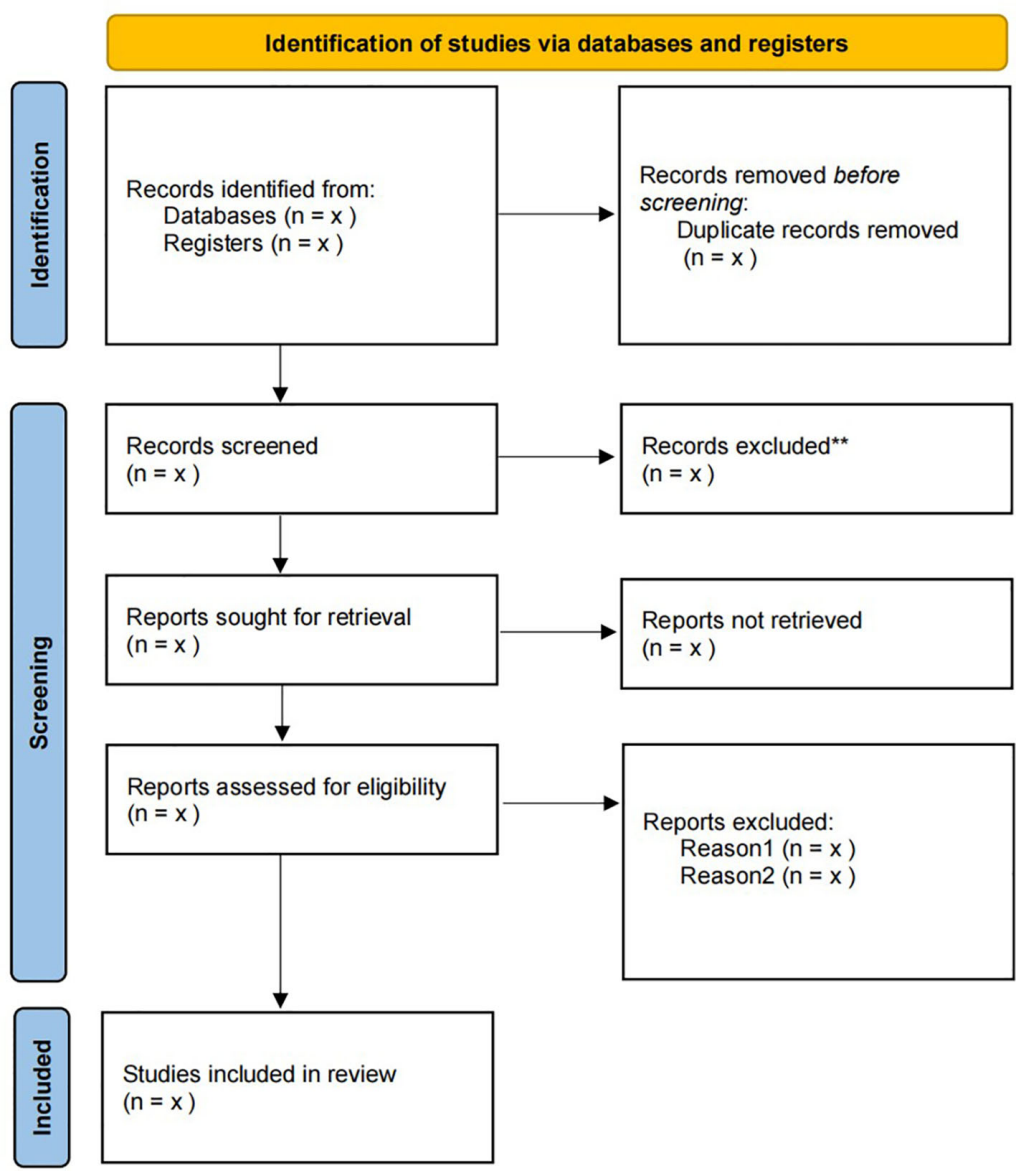
The PRISMA flow diagram of the study selection process.

A standardized data extraction form, developed by two independent researchers (PCZ and JFW), will be utilized to systematically collect study characteristics and outcome measures. In the presence of duplicate data, priority will be given to publications with 1) larger sample sizes and 2) extended follow-up durations to maximize data validity. Consensus for any discrepancies in study selection or data extraction will be achieved through consultation with a third independent investigator (HW). The PRISMA flow diagram detailing the study selection process is provided in **Figure 1**.

### 2.5 Assessment of risk of bias

Two reviewers (XYX and NZ) will independently assess the risk of bias using the Cochrane Risk of Bias Tool Version 2.0 (20). The evaluation will be conducted across five domains: (1) the randomization process, (2) deviations from intended interventions, (3) missing outcome data, (4) measurement of the outcome, and (5) selection of reported results. Each domain will be categorized as “high risk of bias,” “moderate risk of bias (some concerns),” or “low risk of bias.” An overall risk of bias classification for each study will also be determined as high, moderate (some concerns), or low. Any discrepancies will be resolved through discussion with the third investigator (HW).

## 3 Statistical analysis

Intervention efficacy will be quantified by the mean change in RADS-2, CDI, or CDRS-R scores from baseline to endpoint, while safety outcomes will be determined by monitoring the incidence of adverse events. Adolescents are at a critical stage of growth and development. Given that clinical features, symptom severity, and treatment responses may differ significantly depending on the developmental stage, this study will conduct subgroup analyses by age, with the population divided into two groups: 10–13 years old and 14–19 years old.

Heterogeneity across studies will be assessed using the I² statistic, with values exceeding 50% indicating substantial heterogeneity. Potential publication bias will be evaluated through funnel plot asymmetry tests complemented by Egger’s regression analysis.

Bayesian network meta-analysis will be implemented using Markov chain Monte Carlo (MCMC) sampling within a random-effects framework, executed in R statistical software (v4.0.5) via the gemtc package (v0.8-7). Model selection between fixed-effects and random-effects approaches will be guided by deviance information criterion (DIC) comparisons. Convergence of MCMC chains will be validated through Brooks-Gelman-Rubin diagnostics, with potential scale reduction factors (PSRF) <1.1 considered indicative of adequate convergence (21).

### 3.1 Evidence synthesis

The network geometry of treatment comparisons will be graphically represented using a node-edge diagram, with node sizes weighted by participant numbers and edge thickness proportional to the number of direct comparisons. Efficacy outcomes will be synthesized in league tables as posterior mean differences (MDs) with 95% credible intervals (CrIs), whereas safety profiles will be expressed as odds ratios (ORs) accompanied by 95% CrIs. Statistical significance will be interpreted through the exclusion of null values within CrIs (MD CrIs not spanning 0; OR CrIs not encompassing 1). To control multiplicity in hypothesis testing, a family-wise error rate adjustment will be applied (α-level = 0.05) using the Holm-Bonferroni method.

### 3.2 Ranking probabilities

The probability estimate of each acupuncture-related therapy ranking highest in efficacy will be estimated by determining the proportion of Markov chain Monte Carlo (MCMC) iterations in which it demonstrated the largest mean difference (MD) compared to controls. Ranks for second, third, and subsequent positions will be similarly derived.

### 3.3 Inconsistency assessment

Global inconsistency in network meta-analyses will be assessed through the node-splitting approach, specifically applied to closed loops formed by three-intervention comparisons. This method quantifies the discrepancy between direct evidence (head-to-head trials) and indirect evidence (derived via common comparators) using Bayesian *p <*0.05 indicating significant inconsistency.

All the data analysis will be performed using the R statistical software (version 4.2.2).

### 3.4 Certainty of evidence assessment

The certainty of evidence for all outcomes will be evaluated through the Confidence in Network Meta-Analysis (CINeMA)web application (22, 23). The CINeMA includes 6 domains: (1) within-study bias, (2) across-studies bias, (3) indirectness, (4) imprecision, (5) heterogeneity, and (6) incoherence. The overall evidence certainty will ultimately be classified according to the GRADE framework as high, moderate, low, or very low.

## 4 Discussion

Depression is a major contributor to the global burden of disease and is associated with significant personal, societal, and economic costs (24). Adolescent depression has become an increasingly serious issue, with its prevalence rising sharply over the past decade (25, 26). Adolescents diagnosed with depression are at higher risk of developing other mental health disorders, such as anxiety disorders and bipolar affective disorders (27). Symptoms of depression have been found to be associated with eating disturbances and functional somatic symptoms. High-risk behaviors linked to depression in young individuals include increased suicide attempts, elevated alcohol, nicotine, and substance abuse (28–31), as well as physical health issues such as heightened risks of cardiovascular diseases and obesity (32, 33). Therefore, providing appropriate treatment to adolescents with depression is of utmost importance.

Adolescents are at a sensitive and critical stage of both physiological and psychological development, making the treatment strategies for adolescent depression distinct from those for adult depression. Limited evidence suggests that escitalopram, sertraline, and duloxetine may be effective for adolescent depression. Specifically, some adolescent depression patients may not only fail to achieve ideal antidepressant effects when treated with the maximum dosage of medication but may also suffer severe physical harm from drug side effects (34). Therefore, the safety of medications must be strictly controlled in the treatment of adolescent depression, aiming to alleviate symptoms while avoiding any side effects that could exacerbate the condition or cause additional harm.

In recent years, an increasing number of studies have focused on the application of acupuncture in adolescent depression. According to traditional Chinese medical theory, the pathogenesis of depression can be traced to differences in individual constitution, compounded by adverse external stimuli, such as emotional distress and prolonged psychological suppression without adequate expression. Previous study (35) have demonstrated that acupuncture may increase the levels of monoamine neurotransmitters such as serotonin (5-hydroxytryptamine, 5-HT) in the body, thereby exerting an antidepressant effect.

Network meta-analysis (NMA) allows for the comprehensive evaluation of multiple treatment options by synthesizing evidence from both direct and indirect comparisons across diverse studies (36). This study protocol will focus exclusively on research published in either English or Chinese. It is important to note that this language restriction may limit the diversity of the included data and could potentially introduce selection bias. In this NMA, the types of acupuncture therapies included, as well as their combinations, will be explicitly defined. This systematic approach aims to provide a thorough assessment of the efficacy of various acupuncture-based interventions in the management of adolescent depression, thereby offering more robust evidence to inform clinical practice.

This NMA has several limitations. The analysis is restricted to studies published in English or Chinese, which may lead to potential language bias and limit the generalizability of our findings. Additionally, the methodological shortcomings of the included studies, such as potential biases associated with non-blinded outcome assessments and the presence of small sample sizes in individual trials, may compromise the reliability and robustness of our findings. To overcome these limitations, we will address these issues through the following measures: a. Searching international trial registries (e.g., ClinicalTrials.gov) for unpublished data, regardless of language, and contacting authors for English or Chinese summaries; b. Conducting a sensitivity analysis.

## References

[1] GBD 2019 Mental Disorders Collaborators: Global, regional, and national burden of 12 mental disorders in 204 countries and territories, 1990-2019: a systematic analysis for the Global Burden of Disease Study 2019. Lancet Psychiatry. (2022) 9(2):137–50. doi: 10.1016/S2215-0366(21)00395-3, PMID: 35026139 PMC8776563

[2] Goodall J Fisher C Hetrick S Phillips L Parrish EM Allott K . Neurocognitive functioning in depressed young people: A systematic review and meta-analysis. Neuropsychol Rev. (2018) 28:216–31. doi: 10.1007/s11065-018-9373-9, PMID: 29680959

[3] Cuijpers P Miguel C Harrer M Plessen CY Ciharova M Ebert D . Cognitive behavior therapy vs. control conditions, other psychotherapies, pharmacotherapies and combined treatment for depression: a comprehensive meta-analysis including 409 trials with 52,702 patients. World Psychiatry. (2023) 22:105–15. doi: 10.1002/wps.21069, PMID: 36640411 PMC9840507

[4] Christ C Schouten MJ Blankers M van Schaik DJ Beekman AT Wisman MA . Internet and computer-based cognitive behavioral therapy for anxiety and depression in adolescents and young adults: systematic review and meta-analysis. J Med Internet Res. (2020) 22:e17831. doi: 10.2196/17831, PMID: 32673212 PMC7547394

[5] Baune BT Fuhr M Air T Hering C . Neuropsychological functioning in adolescents and young adults with major depressive disorder–a review. Psychiatry Res. (2014) 218:261–71. doi: 10.1016/j.psychres.2014.04.052, PMID: 24851725

[6] Zhou X Hetrick SE Cuijpers P Qin B Barth J Whittington CJ . Comparative efficacy and acceptability of psychotherapies for depression in children and adolescents: A systematic review and network meta-analysis. World Psychiatry. (2015) 14:207–22. doi: 10.1002/wps.20217, PMID: 26043339 PMC4471978

[7] Murphy SE Capitão LP Giles SLC Cowen PJ Stringaris A Harmer CJ . The knowns and unknowns of SSRI treatment in young people with depression and anxiety: efficacy, predictors, and mechanisms of action. Lancet Psychiatry. (2021) 8:824–35. doi: 10.1016/S2215-0366(21)00154-1, PMID: 34419187

[8] Virtanen S Lagerberg T Takami Lageborn C Kuja-Halkola R Brikell I Matthews AA . Antidepressant use and risk of manic episodes in children and adolescents with unipolar depression. JAMA Psychiatry. (2024) 81:25–33. doi: 10.1001/jamapsychiatry.2023.3555, PMID: 37755835 PMC10534997

[9] Dwyer JB Stringaris A Brent DA Bloch MH . Annual Research Review: Defining and treating pediatric treatment-resistant depression. J Child Psychol psychiatry Allied disciplines. (2020) 61:312–32. doi: 10.1111/jcpp.13202, PMID: 32020643 PMC8314167

[10] Strawn JR Mills JA Poweleit EA Ramsey LB Croarkin PE . Adverse effects of antidepressant medications and their management in children and adolescents. Pharmacotherapy. (2023) 43:675–90. doi: 10.1002/phar.2767, PMID: 36651686 PMC10378577

[11] Hsu DT Diehl DL . Acupuncture. The West gets the point. Lancet (London England). (1998) 352 Suppl 4:SIV1. doi: 10.1016/S0140-6736(98)90263-X, PMID: 9872148

[12] Yang X Gong W Ma X Wang S Wang X Guo T . Factor analysis of electroacupuncture and selective serotonin reuptake inhibitors for major depressive disorder: an 8-week controlled clinical trial. Acupunct Med. (2020) 38:45–52. doi: 10.1136/acupmed-2017-011412, PMID: 31544488

[13] Liu H Chen X . The effects of Acupuncture on the Behavior and hippocampal amino acid neurotransmitters in depression model rats. J Med Res. (2010) 39:79–81.

[14] Zhao B Li Z Wang Y Ma X Wang X Wang X . Manual or electroacupuncture as an add-on therapy to SSRIs for depression: A randomized controlled trial. J Psychiatr Res. (2019) 114:24–33. doi: 10.1016/j.jpsychires.2019.04.005, PMID: 31015098

[15] Li W Sun M Yin X Lao L Kuang Z Xu S . The effect of acupuncture on depression and its correlation with metabolic alterations: A randomized controlled trial. Medicine. (2020) 99:e22752. doi: 10.1097/MD.0000000000022752, PMID: 33120777 PMC7581113

[16] Dong Y Huang HW Zhang Y Wang X Yao HX Deng JJ . Clinical Research on the treatment of adolescent depression with Diaodu Tongnao Acupuncture Method. Chinese Med Herald. (2017) 23(02):67–71.

[17] Naing C Reid SA Aung K . Comparing antibiotic treatment for leptospirosis using network meta-analysis: a tutorial. BMC Infect Dis. (2017) 17:29. doi: 10.1186/s12879-016-2145-3, PMID: 28056834 PMC5217240

[18] Shamseer L Moher D Clarke M Ghersi D Liberati A Petticrew M . Preferred reporting items for systematic review and meta-analysis protocols (PRISMA-P) 2015: elaboration and explanation. BMJ (Clinical Res ed). (2015) 350:g7647. doi: 10.1136/bmj.g7647, PMID: 25555855

[19] World Health Organization . Adolescent health (2023). Available online at: https://www.who.int/health-topics/adolescent-health (Accessed June 22, 2025).

[20] Sterne JAC Savović J Page MJ Elbers RG Blencowe NS Boutron I . RoB 2: a revised tool for assessing risk of bias in randomised trials. BMJ (Clinical Res ed). (2019) 366:l4898. doi: 10.1136/bmj.l4898, PMID: 31462531

[21] Jansen JP Crawford B Bergman G Stam W . Bayesian meta-analysis of multiple treatment comparisons: an introduction to mixed treatment comparisons. Value Health. (2008) 11:956–64. doi: 10.1111/j.1524-4733.2008.00347.x, PMID: 18489499

[22] Nikolakopoulou A Higgins JPT Papakonstantinou T Chaimani A Del Giovane C Egger M . CINeMA: An approach for assessing confidence in the results of a network meta-analysis. PloS Med. (2020) 17:e1003082. doi: 10.1371/journal.pmed.1003082, PMID: 32243458 PMC7122720

[23] Papakonstantinou T Nikolakopoulou A Higgins JPT Egger M Salanti G . CINeMA: Software for semiautomated assessment of the confidence in the results of network meta-analysis. Campbell Syst Rev. (2020) 16:e1080. doi: 10.1002/cl2.1080, PMID: 37131978 PMC8356302

[24] GBD 2017 Disease and Injury Incidence and Prevalence Collaborators: Global, regional, and national incidence, prevalence, and years lived with disability for 354 diseases and injuries for 195 countries and territories, 1990-2017: a systematic analysis for the Global Burden of Disease Study 2017. Lancet (London England). (2018) 392(10159):1789–858. doi: 10.1016/S0140-6736(18)32279-7, PMID: 30496104 PMC6227754

[25] Collishaw S . Annual research review: Secular trends in child and adolescent mental health. J Child Psychol psychiatry Allied disciplines. (2015) 56:370–93. doi: 10.1111/jcpp.12372, PMID: 25496340

[26] Patton GC Sawyer SM Santelli JS Ross DA Afifi R Allen NB . Our future: a Lancet commission on adolescent health and wellbeing. Lancet (London England). (2016) 387:2423–78. doi: 10.1016/S0140-6736(16)00579-1, PMID: 27174304 PMC5832967

[27] McLeod GFH Horwood LJ Fergusson DM . Adolescent depression, adult mental health and psychosocial outcomes at 30 and 35 years. psychol Med. (2016) 46:1401–12. doi: 10.1017/S0033291715002950, PMID: 26818194

[28] Campo JV . Annual research review: functional somatic symptoms and associated anxiety and depression–developmental psychopathology in pediatric practice. J Child Psychol psychiatry Allied disciplines. (2012) 53:575–92. doi: 10.1111/j.1469-7610.2012.02535.x, PMID: 22404290

[29] Foley JD Vanable PA Brown LK Carey MP DiClemente RJ Romer D . Depressive symptoms as a longitudinal predictor of sexual risk behaviors among African-American adolescents. Health psychology: Off J Division Health Psychology Am psychol Assoc. (2019) 38:1001–9. doi: 10.1037/hea0000780, PMID: 31380687 PMC6800787

[30] Groenman AP Janssen TWP Oosterlaan J . Childhood psychiatric disorders as risk factor for subsequent substance abuse: A meta-analysis. J Am Acad Child Adolesc Psychiatry. (2017) 56:556–69. doi: 10.1016/j.jaac.2017.05.004, PMID: 28647007

[31] Lehrer JA Shrier LA Gortmaker S Buka S . Depressive symptoms as a longitudinal predictor of sexual risk behaviors among US middle and high school students. Pediatrics. (2006) 118:189–200. doi: 10.1542/peds.2005-1320, PMID: 16818565

[32] Goldstein BI Korczak DJ . Links between child and adolescent psychiatric disorders and cardiovascular risk. Can J Cardiol. (2020) 36:1394–405. doi: 10.1016/j.cjca.2020.06.023, PMID: 32628978

[33] Mannan M Mamun A Doi S Clavarino A . Prospective associations between depression and obesity for adolescent males and females- A systematic review and meta-analysis of longitudinal studies. PloS One. (2016) 11:e0157240. doi: 10.1371/journal.pone.0157240, PMID: 27285386 PMC4902254

[34] Hetrick SE McKenzie JE Bailey AP Sharma V Moller CI Badcock PB . New generation antidepressants for depression in children and adolescents: a network meta-analysis. Cochrane Database Syst Rev. (2021) 5:CD013674. doi: 10.1002/14651858.CD013674.pub2, PMID: 34029378 PMC8143444

[35] Zhou XF Li Y Zhou ZH Pan SH . Clinical Observation of Acupuncture in the Treatment of Depression and its effect on Serum Serotonin. Chin Acupuncture Moxibustion. (2015) 35(02):123–6. 25854015

[36] Caldwell DM Ades AE Higgins JPT . Simultaneous comparison of multiple treatments: combining direct and indirect evidence. BMJ (Clinical Res ed). (2005) 331:897–900. doi: 10.1136/bmj.331.7521.897, PMID: 16223826 PMC1255806

