# Supplementary material for: Comparative efficacy and safety of acupuncture for adolescent depression: protocol for a systematic review and Bayesian network meta-analysis
Source: Front Psychiatry. 2025 Jul 23;16:1624825. doi: 10.3389/fpsyt.2025.1624825 (PMC12329222; doi:10.3389/fpsyt.2025.1624825)
Supplement: Supplementary file 1 [file Supplementaryfile1.docx]

**S1. PRISMA-P (Preferred Reporting Items for Systematic review and Meta-Analysis Protocols) 2015 checklist: recommended items to address in a systematic review protocol***

| Section and topic | Item No | Checklist item |  |
| --- | --- | --- | --- |
| ADMINISTRATIVE INFORMATION | | | Page |
| Title: |  |  |  |
| Identification | 1a | Identify the report as a protocol of a systematic review | Title page |
| Update | 1b | If the protocol is for an update of a previous systematic review, identify as such | NA |
| Registration | 2 | If registered, provide the name of the registry (such as PROSPERO) and registration number | Abstract part |
| Authors: |  |  |  |
| Contact | 3a | Provide name, institutional affiliation, e-mail address of all protocol authors; provide physical mailing address of corresponding author | Title page |
| Contributions | 3b | Describe contributions of protocol authors and identify the guarantor of the review | Authors’ contributions part |
| Amendments | 4 | If the protocol represents an amendment of a previously completed or published protocol, identify as such and list changes; otherwise, state plan for documenting important protocol amendments | NA |
| Support: |  |  |  |
| Sources | 5a | Indicate sources of financial or other support for the review | Funding part |
| Sponsor | 5b | Provide name for the review funder and/or sponsor | Funding part |
| Role of sponsor or funder | 5c | Describe roles of funder(s), sponsor(s), and/or institution(s), if any, in developing the protocol | Funding part |
| INTRODUCTION | | |  |
| Rationale | 6 | Describe the rationale for the review in the context of what is already known | Introduction part |
| Objectives | 7 | Provide an explicit statement of the question(s) the review will address with reference to participants, interventions, comparators, and outcomes (PICO) | Introduction part |
| METHODS | | |  |
| Eligibility criteria | 8 | Specify the study characteristics (such as PICO, study design, setting, time frame) and report characteristics (such as years considered, language, publication status) to be used as criteria for eligibility for the review | Methods part |
| Information sources | 9 | Describe all intended information sources (such as electronic databases, contact with study authors, trial registers or other grey literature sources) with planned dates of coverage | Methods part |
| Search strategy | 10 | Present draft of search strategy to be used for at least one electronic database, including planned limits, such that it could be repeated | Methods part Supplementary Table 2 |
| Study records: |  |  |  |
| Data management | 11a | Describe the mechanism(s) that will be used to manage records and data throughout the review | Methods part |
| Selection process | 11b | State the process that will be used for selecting studies (such as two independent reviewers) through each phase of the review (that is, screening, eligibility and inclusion in meta-analysis) | Methods part Supplementary Fig. 1 |
| Data collection process | 11c | Describe planned method of extracting data from reports (such as piloting forms, done independently, in duplicate), any processes for obtaining and confirming data from investigators | Methods part |
| Data items | 12 | List and define all variables for which data will be sought (such as PICO items, funding sources), any pre-planned data assumptions and simplifications | Methods part |
| Outcomes and prioritization | 13 | List and define all outcomes for which data will be sought, including prioritization of main and additional outcomes, with rationale | Methods part |
| Risk of bias in individual studies | 14 | Describe anticipated methods for assessing risk of bias of individual studies, including whether this will be done at the outcome or study level, or both; state how this information will be used in data synthesis | Methods part |
| Data synthesis | 15a | Describe criteria under which study data will be quantitatively synthesised | Statistical analysis part |
|  | 15b | If data are appropriate for quantitative synthesis, describe planned summary measures, methods of handling data and methods of combining data from studies, including any planned exploration of consistency (such as I^2^, Kendall’s τ) | Statistical analysis part |
|  | 15c | Describe any proposed additional analyses (such as sensitivity or subgroup analyses, meta-regression) | Statistical analysis part |
|  | 15d | If quantitative synthesis is not appropriate, describe the type of summary planned | Statistical analysis part |
| Meta-bias(es) | 16 | Specify any planned assessment of meta-bias(es) (such as publication bias across studies, selective reporting within studies) | Statistical analysis part |
| Confidence in cumulative evidence | 17 | Describe how the strength of the body of evidence will be assessed (such as GRADE) | Statistical analysis part |

**NA: not applicable, NS: not stated.**

*** It is strongly recommended that this checklist be read in conjunction with the PRISMA-P Explanation and Elaboration (cite when available) for important clarification on the items. Amendments to a review protocol should be tracked and dated. The copyright for PRISMA-P (including checklist) is held by the PRISMA-P Group and is distributed under a Creative Commons Attribution Licence 4.0.**

*From: Shamseer L, Moher D, Clarke M, Ghersi D, Liberati A, Petticrew M, Shekelle P, Stewart L, PRISMA-P Group. Preferred reporting items for systematic review and meta-analysis protocols (PRISMA-P) 2015: elaboration and explanation. BMJ. 2015 Jan 2;349(jan02 1):g7647.*

# **S2: Search Strategy**

**I. PubMed**

#1 Search (“depression” [MeSH Terms])

#2 Search (“depression” [Title/Abstract] OR “depressive disorder” [Title/Abstract] OR “depressive”[Title/ Abstract] OR “Depressive Symptoms“[Title/ Abstract])

#3 #1 or #2

#4 Search (“acupuncture”[MeSH Terms])

#5 Search (“acupuncture”[Title/Abstract] OR “electro-acupuncture” [Title/Abstract] OR “warming needling” [Title/Abstract] OR “fire needling”[Title/Abstract] OR “bloodletting”[Title/Abstract] OR “auriculo-acupuncture”[Title/Abstract] OR “auricular” [Title/Abstract] OR “moxibustion” [Title/Abstract] OR, “cupping” [Title/Abstract]OR “acupoint*” [Title/Abstract])

#6 #4 or #5

#7 Search (“adolescent”[MeSH Terms]

#8 “adolescent” [Title/Abstract] OR “teenager” [Title/Abstract] OR “juvenile” [Title/Abstract] OR “young” [Title/Abstract] OR “youngsters”[Title/Abstract] OR “student”[Title/Abstract]

#9 #7 or #8

#10 Search (“randomized controlled trial” [Title/Abstract] OR “controlled clinical “[Title/Abstract] OR “trial “[Title/Abstract] OR “group”[Title/Abstract] OR “placebo” [Title/Abstract] OR “randomly” [Title/Abstract])

#11 #3 and #6 and #9 and #10

**II. Cochrane Central Register of Controlled Trials (CENTRAL)**

1. MeSH descriptor: [depression] explode all trees
2. ((depression or depressive or depressive disorder or Depressive Symptoms) near/3 (despondent * or depressed)):ti,ab
3. #1 or #2
4. MeSH descriptor: [acupuncture] explode all trees
5. （acupuncture therapy or acupoint* or meridian* or electroacupuncture or electro-acupuncture or acupressure* or community or fire needling or warming needling or stimulation or education* or counsel* or 8. “acup* or point* ”or “mox*”or “needl*” or “ auriculo-acup*” or “auricular” or “auricular point” or “ear” or “Ear point sticking” or “auricular acupressure” or “cup*” or “bloodlet*” or “Ear Massage or. “auricular-plaster therapy” or “EA” or “scalp acupuncture” or “Head acupuncture”）:ti,ab,kw
6. #4 or #5
7. MeSH descriptor: [adolescent] explode all trees
8. （adolescent or teenager orstudent* or juvenile or young or youngster *）:ti,ab,kw
9. #7 or #8
10. randomized controlled trial: ti,ab,kw (Word variations have been searched)
11. High blood pressure:ti,ab,kw (Word variations have been searched)
12. controlled clinical trial:ti,ab,kw (Word variations have been searched)
13. randomly:ti,ab,kw (Word variations have been searched)
14. placebo:ti,ab,kw (Word variations have been searched)
15. #10 or #11 or #12 or #13 or #14
16. #3 and #6 and #9 and #15

**III. EMBASE**

1. exp depression /

2. exp depressive disorder /

3. exp depressive* /

4. exp despondent* /

5. exp depressed* /

6. or/1-5

7. exp Acupuncture/

8. exp Acupuncture Therapy/

9. (acupuncture or acupoint* or meridian*). ti,ab.

10. (electroacupuncture or electro-acupuncture). ti,ab.

11. (fire needling or warming needling or acupressure* or acup* point* or mox* or needl* or auriculo-acup* or cup* or bloodlet*). ti,ab.

12. or/7-11

13. exp adolescent /

14. exp teenager /

15. (adolescent or teenager orstudent* or juvenile or young or youngster *）:ti,ab

16 or/13-15

17. randomized controlled trial. ti,ab.

18. controlled clinical trial.ti,ab.

19. randomi$.ti,ab.

20. randomly.ti,ab.

21. trial.ti,ab.

22. group.ti,ab.

23. placebo.ti,ab.

24. or/17-23

25. 6 and 12 and 16 and 24

**IV. Web of Science**

Indexes=SCI-EXPANDED, SSCI, A&HCI, ESCI Timespan=All years

1. TOPIC: ((depression OR depressed* OR depressive* OR Depressive Symptoms ) NEAR/3 (depressive disorder))
2. TOPIC: ((acupuncture OR acupuncture therapy OR electroacupuncture OR electro-acupuncture OR fire needling OR warming needling) NEAR/3 (acupressure* or acup* point* or mox* or needl* or auriculo-acup* or cup* or bloodlet*))
3. TOPIC: ((adolescent OR teenager OR juvenile) NEAR/3 (student* or young or youngster*))
4. TS= clinical trial* OR TS= randomly OR TS=controlled trial* OR TS=prospective stud* OR TS=random* OR TS=placebo* OR TS=(single blind*) OR TS=(double blind*)
5. #1 AND #2 AND #3 AND #4

**V. The China National Knowledge Infrastructure (CNKI) databases**

1. (SU= ‘depression’ or SU= ‘depressed*’ or SU= ‘depressive disorder’ or SU= ‘Depressive Symptoms’ or SU= ‘Emotional’ or SU= ‘Emotional Depression’)

2. (SU= ‘adolescent*’ or SU= ‘teenager*’ or SU= ‘juvenile’ or SU= ‘student*’ or SU= ‘young’ or SU= ‘youngster*’ SU= ‘Teen*’ or SU= ‘Youth*’)

3. (SU= ‘acupuncture’ or SU= ‘electroacupuncture’ or SU= ‘electro-acupuncture’ or SU= ‘acupuncture therapy’ or SU= ‘fire needling’ or SU= ‘warming needling’ or SU= ‘acupressure*’ or SU= ‘acup* point*’ or SU= ‘mox*’ or SU= ‘auriculo-acup*’ or SU= ‘warming needling’ or SU= ‘cup*’ or SU= ‘bloodlet*’ or SU= ‘meridian*’)

4. (SU= ‘clinical trial*’ or SU= ‘randomly’ or SU= ‘controlled trial*’ or SU= ‘prospective stud*’ or SU= ‘random*’ or SU= ‘placebo*’ or SU= ‘single blind*’ or SU= ‘double blind*’ or SU= ‘trial’ or SU= ‘group’)

5. #1 and #2 and #3 and #4
